# The Accuracy Comparison of Three Simultaneous Localization and Mapping (SLAM)-Based Indoor Mapping Technologies †

**DOI:** 10.3390/s18103228

**Published:** 2018-09-25

**Authors:** Yuwei Chen, Jian Tang, Changhui Jiang, Lingli Zhu, Matti Lehtomäki, Harri Kaartinen, Risto Kaijaluoto, Yiwu Wang, Juha Hyyppä, Hannu Hyyppä, Hui Zhou, Ling Pei, Ruizhi Chen

**Affiliations:** 1 Centre of Excellence in Laser Scanning Research, Finnish Geospatial Research Institute (FGI), Geodeetinrinne 2, FI-02431 Kirkkonummi, Finland; Yuwei.chen@nls.fi (Y.C.); tangjian@whu.edu.cn (J.T.); lingli.zhu@nls.fi (L.Z.); matti.lehtomaki@nls.fi (M.L.); harri.kaartinen@nls.fi (H.K.); risto.kaijaluoto@nls.fi (R.K.); sindywyw@hotmail.com (Y.W.); juha.hyyppa@nls.fi (J.H.); hannu.hyyppa@aalto.fi (H.H.); 2 GNSS Research Center, Wuhan University, 129 Luoyu Road, Wuhan 430079, China; 3 School of Automation, Nanjing University of Science and Technology, Nanjing 210094, China; 4 Department of Built Environment, Aalto University, P.O. Box 15800, 00076 AALTO, Finland; 5 Electronic Information School, Wuhan University, 129 Luoyu Road, Wuhan 430079, China; zhouhui@whu.edu.cn; 6 Shanghai Key Laboratory of Navigation and Location-Based Services, School of Electronic Information and Electrical Engineering, Shanghai Jiao Tong University, Shanghai 200240, China; ling.pei@sjtu.edu.cn; 7 State Key Laboratory of Information Engineering in Surveying, Mapping and Remote Sensing, Wuhan University, 129 Luoyu Road, Wuhan 430079, China; ruizhi.chen@whu.edu.cn

**Keywords:** indoor mapping, SLAM, LiDAR, accuracy comparison, mobile mapping, terrestrial mapping

## Abstract

The growing interest and the market for indoor Location Based Service (LBS) have been drivers for a huge demand for building data and reconstructing and updating of indoor maps in recent years. The traditional static surveying and mapping methods can’t meet the requirements for accuracy, efficiency and productivity in a complicated indoor environment. Utilizing a Simultaneous Localization and Mapping (SLAM)-based mapping system with ranging and/or camera sensors providing point cloud data for the maps is an auspicious alternative to solve such challenges. There are various kinds of implementations with different sensors, for instance LiDAR, depth cameras, event cameras, etc. Due to the different budgets, the hardware investments and the accuracy requirements of indoor maps are diverse. However, limited studies on evaluation of these mapping systems are available to offer a guideline of appropriate hardware selection. In this paper we try to characterize them and provide some extensive references for SLAM or mapping system selection for different applications. Two different indoor scenes (a L shaped corridor and an open style library) were selected to review and compare three different mapping systems, namely: (1) a commercial Matterport system equipped with depth cameras; (2) SLAMMER: a high accuracy small footprint LiDAR with a fusion of hector-slam and graph-slam approaches; and (3) NAVIS: a low-cost large footprint LiDAR with Improved Maximum Likelihood Estimation (IMLE) algorithm developed by the Finnish Geospatial Research Institute (FGI). Firstly, an L shaped corridor (2nd floor of FGI) with approximately 80 m length was selected as the testing field for Matterport testing. Due to the lack of quantitative evaluation of Matterport indoor mapping performance, we attempted to characterize the pros and cons of the system by carrying out six field tests with different settings. The results showed that the mapping trajectory would influence the final mapping results and therefore, there was optimal Matterport configuration for better indoor mapping results. Secondly, a medium-size indoor environment (the FGI open library) was selected for evaluation of the mapping accuracy of these three indoor mapping technologies: SLAMMER, NAVIS and Matterport. Indoor referenced maps were collected with a small footprint Terrestrial Laser Scanner (TLS) and using spherical registration targets. The 2D indoor maps generated by these three mapping technologies were assessed by comparing them with the reference 2D map for accuracy evaluation; two feature selection methods were also utilized for the evaluation: interactive selection and minimum bounding rectangles (MBRs) selection. The mapping RMS errors of SLAMMER, NAVIS and Matterport were 2.0 cm, 3.9 cm and 4.4 cm, respectively, for the interactively selected features, and the corresponding values using MBR features were 1.7 cm, 3.2 cm and 4.7 cm. The corresponding detection rates for the feature points were 100%, 98.9%, 92.3% for the interactive selected features and 100%, 97.3% and 94.7% for the automated processing. The results indicated that the accuracy of all the evaluated systems could generate indoor map at centimeter-level, but also variation of the density and quality of collected point clouds determined the applicability of a system into a specific LBS.

## 1. Introduction

The recent widespread use of smartphones equipped with different miniature navigation and positioning sensors [1] aiming to develop future Indoor Location-Based Services (InLBS) has also boosted new indoor mapping technologies. Smartphone-based solutions, sensors and algorithms have been investigated to enhance indoor positioning accuracy [2,3,4,5,6,7,8]. The search for an efficient, low-cost high accuracy indoor mapping technology in establishing a framework for LBS has been challenging. Indoor surveying mapping is a complicated task [9] due to the complexity of indoor spatial structures and the lack of global references. The indoor space is fragmented to rooms, corridors, stairs and facilities with diverse shapes and functions, and distributed in a three dimensional space. The characteristics to public buildings, such as in airports, hospitals and supermarkets, is that the spatial extent of the indoor environment may be enormous. Bustling with human activity they pose a challenge in regard to mapping results; the layout of the indoor environment can change frequently and moving objects necessitate outlier detection robustness. Global Navigation Satellite System (GNSS) signals cannot be applied to obtain absolute positions due to Radio Frequency (RF) signal blocking, thus, conventional GNSS/Inertial Navigation System (INS)- based mobile mapping techniques may not be applied indoors. Typically, centimeter level mapping accuracy is required for indoor data for subsequent applications, for example for real estate management and development, as well as emergency services possibly relying on such data. The use of stationary total-station based surveying and mapping can be applied to obtain accurate indoor mapping results, but it is a labor-intensive interactive method with low efficiency for comprehensive indoor mapping and map/model updating. Simultaneous Location and Mapping (SLAM) has been shown by the research community to be a promising technology for indoor mapping in. It usually operated with photographic sensors like cameras, ranging sensors like radar, LiDAR or depth cameras like the Microsoft Kinect [10,11,12,13,14,15,16]. According to the sensors employed, it can be categorized as visual SLAM, LiDAR SLAM and RGBD SLAM, which are the three most widely used SLAM techniques.

Visual SLAM is implemented by utilizing visual features as landmarks for pose estimation. Many related works have been published on variants if this technique, for instance Parallel Tracking and Mapping (PTAM), SVO-SLAM, event-camera SLAM et al. PTAM is one of the implementations which firstly introduces the idea of splitting camera tracking and mapping and running them simultaneously with two threads [17]. SVO-SLAM is a semi-direct visual odometry algorithm that is precise, robust, and faster than current state-of-the-art methods [18,19]. The algorithm operates directly on pixel intensities not responding to features. It is expected to be more robust in motion blur or no-texture environments. Recently, an event camera sensor was developed and applied to SLAM [20,21]. An event camera is a silicon retina which outputs not a sequence of video frames like a standard camera, but a stream of asynchronous spikes, each with pixel location, sign and precise timing, indicating when individual pixels record a threshold and log intensity change. Compared with conventional cameras, some remarkable advances can be obtained, which are covered in detail in [20,21].

LiDAR SLAM is mainly based on LiDAR sensors. LiDAR is a ranging sensor which can provide point clouds to construct a map. Many researchers have utilized it to develop SLAM including 2D and 3D [22,23,24,25,26,27,28]. Feature matching algorithms include the classical Iterative Closest Point (ICP) [22] and its extended variations Iterative Closest Line (ICL) [23] and Iterative Closest Plane (ICP) [24] and gird map-based Maximum Likelihood Estimation (MLE) have been widely applied in SLAM mapping systems [25,26,27]. A variety of laser scanners featuring different ranging accuracies and resolutions (such as FARO, Velodyne, Sick LMSx, Hokuyo and UTM-x) have been commonly used in SLAM solutions [28].

RGB-D is a sort of sensor providing RGB images with depth information, which is to some extent a combination of a camera and a LiDAR sensor. Like visual SLAM, RGB-D SLAM is an iterative process, which can be divided into five steps: (1) feature detection, (2) descriptor extraction, (3) feature matching, (4) motion estimation and (5) motion transformation optimization. Firstly, two acquired RGB-D images are processed usually SIFT or SURF algorithms for features detecting and describing [29,30,31,32,33,34,35,36,37,38,39]. Thereby, pairs of matching features are collected and obtained. From each pair of matching points, depth information is included to generate 3D matching points, which are employed for motion estimation.

As mentioned before, there are various SLAM algorithms supported by different technical means. However, this makes it difficult to select the best solution for a given application with a limited budget. Preceding works have concentrated on the development of indoor mapping technologies, whereas this contribution/study is an attempt to answer how well SLAM-based mapping systems perform for indoor mapping with different system configurations. Also, what kind of feature selection criteria should be followed for a particular sensor and the SLAM algorithm has still not thoroughly investigated before. Thereby, in this paper, we preliminarily investigate this issue by comparing three SLAM-based indoor mapping systems. The first system to be evaluated in the comparison is Matterport, which is a commercial indoor mapping system. It is evaluated because it provides an interesting low-cost indoor mapping solution, and to our best knowledge, it has not been evaluated before in a published work. The second one is the SLAMMER, equipped with a high accurate small footprint LiDAR—the Faro Focus3D 120S—and based on an open source Hector-SLM algorithm (a variant of the grid map-based MLE algorithm) used in combination with a Hector SLAM back-end to generate 2D indoor point clouds. The third system is NAVIS, which is another SLAM system self-developed by FGI based on a low-cost large-footprint Hokuyo UTM-30LX-EW laser scanner as the ranging sensor and the IMLE (another variation of grid map based MLE) as the SLAM scan matching algorithm [40,41,42,43,44,45,46,47,48,49,50].

Two different scenes were selected for evaluating the aforementioned SLAM technologies. The first scene is an L-shaped corridor (on the 2nd floor of FGI) with a length of approximately 80 m which is selected as the test field for a commercial depth sensor-based Matterport SLAM. We attempt to characterize the advantages and disadvantages of this mapping technique by comparing each indoor mapping results from different scanning configuration with the referenced map. For the field test of the first scene, several questions are investigated in parallel:

(1) Whether the mapping error is propagated or not for Matterport sensor since the sensor is generally considered as a SLAM-based mapping sensor;
(2) Whether the mapping trajectory of Matterport will affect the final mapping results;
(3) Whether there is any optimal operation configuration of Matterport that can offer better indoor mapping results.

Secondly, the performance evaluations of the mapping accuracy of these three indoor mapping technologies are conducted in the FGI open style library. Corner points of rectangular objects (heavy bookshelves and cabinets) are utilized as features of comparison. If the coordinates of the corner points of rectangular objects can be extracted, more feature parameters such as length and size of objects could also be calculated, and used for evaluations. Two corner feature extraction methods are used in the research: interactive selection and minimum bounding rectangles (MBRs) selection. The evaluation results based on features from the two selection methods are then analyzed. Indoor reference maps for both scenes are generated with a small footprint Terrestrial Laser Scanner (TLS), using spherical registration targets and collecting multiple scans in different positions within the corridor and library. The remainder of this paper is organized as follows: Section 2 gives a brief introduction of the three indoor mapping systems involved in this study, Section 3 describes the test site characteristics and accuracy comparing methods utilized in the research; the experimental results are discussed in Section 4; and Section 5 draws conclusions from the study. This paper is the extension of our conference paper “SLAM Based Indoor Mapping Comparison: Mobile or Terrestrial?”, which is accepted and presented by IEEE Ubiquitous Positioning, Indoor Navigation and Location-Based Services 2018 [50].

## 2. System Overview

### 2.1. Matterport

**Figure 1:**
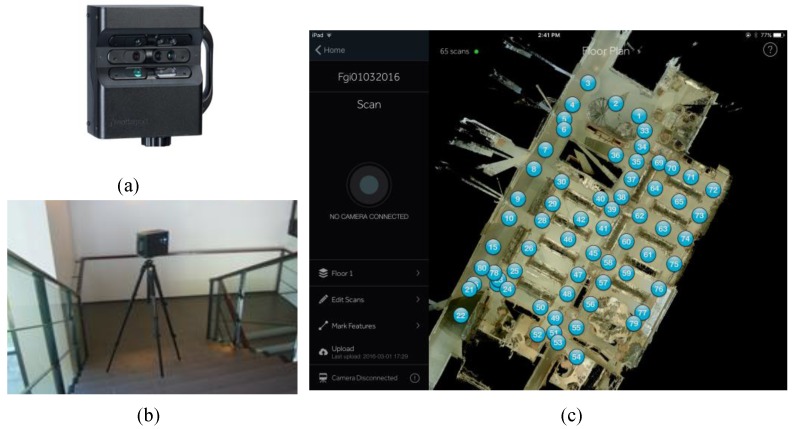
(**a**) Matterport 3D camera; (**b**) Matterport in operation for mapping FGI indoor environment; (**c**) Top view of a 3D model created by Matterport with multiple scans from the test site. The number of needed scans is illustrated in the image.

As is shown in Figure 1a, the Matterport 3D camera uses the same depth sensor as the Microsoft Kinect to quickly capture the appearance and dimensions of a space and create its digital spatial model. The camera consists of an array of three sensors and a motor that revolves the camera 360 degrees to capture the data needed to create a 3D model of an indoor space. Matterport works, as Figure 1b presents, by standing on a horizontal tripod and scanning the nearby environment. It estimates interior dimensions and captures objects, colors and textures by repeated scanning from multiple positions within the mapping area with maximum 12 feet distance between any two neighboring scan stations [44]. The camera is cost effective, and an untrained operator can produce consistent 3D models with texture. Point cloud data can be generated from the constructed 3D models, as shown in Figure 1c. Thus, the 2D map generating mechanism of Matterport is different from that of the two other sensors investigated in this study.

### 2.2. SLAMMER

**Figure 2:**
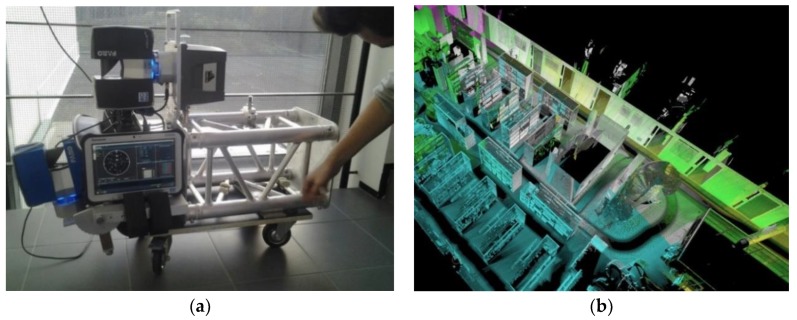
(**a**) SLAMMER platform; (**b**) SLAMMER derived 3D point cloud from the test site could capture the interiors in high level of detail.

Figure 2a presents the system configuration of SLAMMER. SLAMMER hardware consists of a tactical grade IMU (SPAN UIMU-LCI, NovAtel, Calgary, AB, Canada), a horizontally mounted laser scanner (Focus3D 120S, FARO, Lake Mary, FL, US) for SLAM, and a secondary laser scanner (Focus3D X330, FARO, Lake Mary, FL, USA) installed tilted 10 degrees to vertical for 3D point cloud generation when the trajectory of the cart platform is solved with SLAM. A tablet computer is used for recording the IMU and synchronization data by TTL level trigger pulse sent from laser scanner for post-processing. The system antenna has to be placed at a location where GNSS signal is available during the initialization, because the employed clock and IMU need GNSS signal for initialization and aligning. After the initialization, an operator drives the cart to capture the 2D/3D map along the driven path, Figure 2b presents an example of 3D point cloud from the open style library of FGI. IMU is used only for correcting the small roll and pitch movements caused by low quality wheels and for correcting for the rotational movement accrued during a scan, SLAMMER can also be operated without the IMU with slightly lower accuracy. SLAMMER data is currently post-processed: the post-processing time for the library data was approximately 2 h. Most of the time spent is used to search the best possible matches for loop closure by the optimizing backend. Accurate detection of loop closures helps to mitigate error accumulated during scan matching [25]. The processing pipeline for SLAMMER is built on ROS [34] framework and among other uses, ROS is aimed for real time use by mobile robots. In their original state the hector slam and open karto algorithms are meant to be run in real time and as the used pipeline is based on them, it could also be made to run on real time with small modifications (and reduced accuracy). In addition, technical specifications of SLAMMER are listed in Table 1.

**Figure 3:**
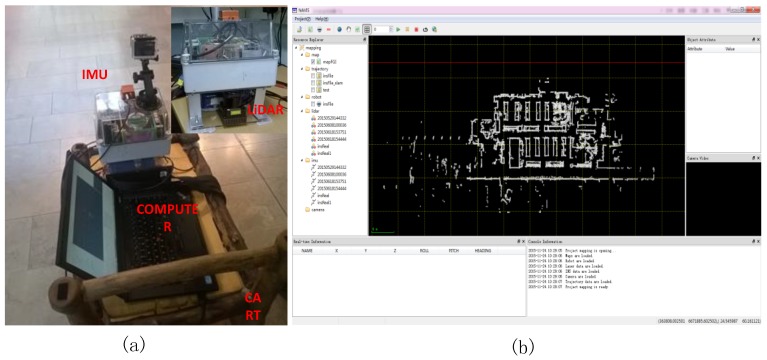
(**a**) NAVIS indoor mapping hardware platform; (**b**) corresponding point cloud from the test site.

As shown in Figure 3a, NAVIS is a Microelectromechanical System (MEMS)-based IMU aided SLAM mapping system developed by FGI. A commercial grade Xsens MTi-G IMU (Xsens, Enschede, Overijssel, Netherlands) and a low-cost, large footprint laser scanner (UTM-30LX-EW, Hokuyo, Tokyo, Japan) were installed on a rigid platform and horizontally carried by a cart [48]. The IMU is connected to a laptop via a USB port and the laser scanner measurements are archived on the laptop via Ethernet. The technical specifications of NAVIS are listed in Table 2. The Xsens MTi-G is a MEMS-type six Degree of Freedom (DOF) miniature IMU with a gyroscope and accelerometer bias instability of 10 degree/h and 2000 mGal [36]. Compared with SLAMMER, the ranging accuracy of the applied Hokuyo laser scanner (3 cm) is a decade less than that of the FARO (2 mm). Self-developed software for recording the raw data and data-processing navigation is programmed with C++ and Qt. The laser scanner is synchronized to the IMU by time stamp of the recording computer [47]. The indoor mapping procedure is similar as SLAMMER; however the IMU needn’t to be initialized outdoors. The NAVIS data is then at local coordinate system, as is SLAMMER data.

In NAVIS, the IMU measurements are utilized to compensate the accumulated drift of SLAM. NAVIS can also be upgraded to generate 3D point cloud by adding one vertically installed laser scanner like the system configuration of the SLAMMER. The mapping processing of NAVIS can be either in real time or post-processed. The operator can observe real time results for monitoring the progress of the mapping. The algorithms applied to NAVIS are able to detect and filter out the dynamic objects such as pedestrians and moving cars (e.g., mapping of indoor parking) during the mapping with a likelihood grid voting algorithm [40].

## 3. Material and Methods

### 3.1. The First Test: An L Corridor

The L-shaped corridor of the 2nd floor of the FGI main building was selected as the test field for characterizing the Matterport SLAM system. The length of each corridor wing is approximate 40 m which is long enough to examine the head estimation error of SLAM at corner points. No loop circle is utilized in this research to focus on the mapping results without the enhancement of the embedded loop closure algorithm in different SLAM systems.

The reference 2D map is collected by a Terrestrial Laser Scanner (TLS; Focus3D 330X, FARO, Lake Mary, FL, USA) with multiple scans from 32 different positions on the floor, and in this paper, we named the TLS scan the T0 test. The angular resolution of the scan data was 3.333 mRad giving thus 33 mm point spacing at 10 m range from the scanner; the range accuracy of TLS is ±2 mm, which implies that a detailed and precise indoor map is generated as reference. The individual scans collected are mutually registered for a complete floor wide point cloud using 199 mm diameter reference targets detected from the data with automated tools of FARO Scene software. The standard deviation of mutually matching is approximate 6–7 mm resulting in the accuracy of the reference 2D being better than 1 cm. Point cloud data generation of Matterport is different with other LiDAR-based indoor mapping solutions. The sensor firstly constructs 3D models of the indoor environment by matching the terrestrial scanning results from multiple positions indoors with maximum 12 feet distance between neighboring scan stations. Then point cloud data can be generated from the constructed 3D models, and thereby, the 2D indoor map can be obtained from the generated point cloud.

### 3.2. The Secnond Test: Three SLAM Based Indoor Mapping Solutions Comparison

**Figure 4:**
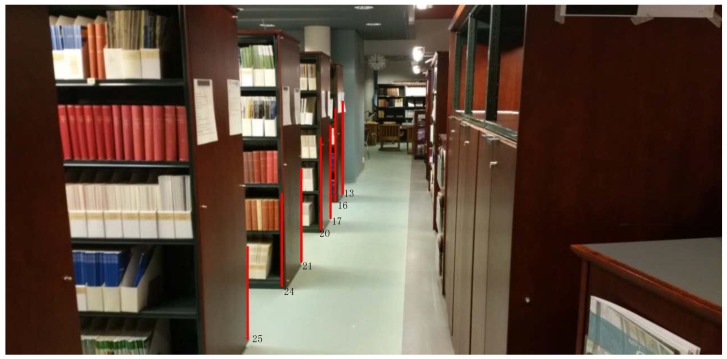
The FGI open style library environment and a part of selected feature points.

The data collection for the tests is carried out in the FGI open style library on the second floor of the building. Figure 4 illustrates the conditions of the library. The size of the area is about 13 × 30 m. The unmovable corners of book shelves, cabinets and walls are the main key points for accuracy evaluation (see the marked red line and the points ID in Figure 4).

The reference 2D map is collected by a TLS laser scanner (Focus3D 330X, FARO, Lake Mary, FL, USA) with multiple scans from 21 different positions in the library. The individual scans collected are mutually registered for a complete floor wide point cloud using same method described in Section 3.1.

**Figure 5:**
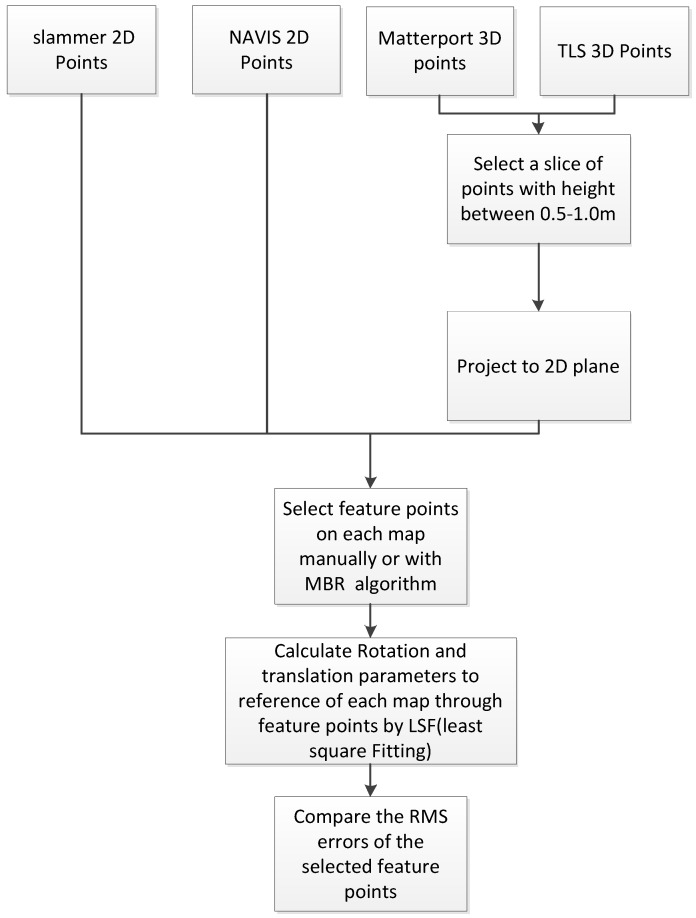
Data process flow for accuracy comparison.

Figure 5 shows the details of the data process flow for accuracy comparison on open style library scene. The outputs of Matterport and TLS system are 3D points cloud, therefore, point slices of height between 0.5 and 1.0 m from the floor plane are chosen from each 3D points cloud for generating 2D maps. Those points are then projected on to a horizontal plane to create a 2D map for comparison purposes. Next, corner points of objects are selected from each map as features to be compared. The comparison is then straightforward. The feature points selected from the TLS reference map are utilized as reference network to evaluate the accuracy by comparing the coordinates of its counterpart in the maps generated by the three different indoor mapping means. In this research, we adopt two feature points selecting methods for comparison: interactive selection and minimum bounding rectangles (MBRs) algorithm.

#### 3.2.1. Feature Point Selection Method

The major reason we use interactive selection for feature point extraction is that the point cloud generated by Matterport is two orders of magnitude sparser than LiDAR-based SLAM and it is hard to correctly extract all feature points with the MBR algorithm for a reliable assessment. During the interactive selection, it might introduce extra noise with misunderstanding of the distribution of the point cloud or mitigate the mapping error by utilizing the expertise of the operator unconsciously.

To more fairly evaluate the mapping accuracy, MBRs based method is also developed and investigated the maximum possible extent to reduce the risk of bias in the evolution result and to detect the feature points semi-automatically base on the premise that all feature points are the corners of rectangle objects:

(1) Firstly, interactively segmentation is adopted to select the “area of interest”. Only the point cloud within the area of interest will be used for matching processing. The major reason for utilizing manually segmentation for indoor laser scanning was the accuracy demand of the results. Certain number of “false” point cloud was generated because of reflection of indoor glass-like objects, such as glass of a book cabinet, or polished metal surfaces.
(2) A MBR algorithm was utilized to detect the rectangle shape within each area of interest.
(3) The corner of rectangle can be extracted from the matched result. The extracted corners were then re-examined by an outlier detector to filter out the mismatched results and honestly evaluate the mapping accuracy, and the threshold of the outlier detector is 0.1 m.

Since most of the objects in the test area were rectangular book shelves, a large number of corners could be extracted by fitting MBRs to the objects. First, objects were segmented interactively from the point clouds of the different systems and TLS. Then, MBRs were fitted to the segments and the coordinates of the corners of the MBRs were retrieved and selected as feature points. An MBR for a 2D point set is defined as the smallest rectangle that encloses the set. The sides of the MBR do not need to be aligned with the coordinate axes and, therefore, a point set can have an infinite number of different MBRs corresponding to different orientations. In the MBR fitting, we decided to choose the orientation, which resulted in a minimum area inside the rectangle. We varied the orientation linearly between 0 and 180 degrees and tried 500 different orientations; therefore, the accuracy of the orientation of the fitted MBR was 0.36 degrees. The corners of the MBR have to be optimized to coincide with the actual corners. The reason for the optimization is the large footprint of the laser beam and other measurement errors that might shift the edges of an object. This increases the size of the object in the point cloud. Therefore, it may be beneficial to shrink the MBRs such that a certain percentage of the outermost points are left outside the rectangle. However the optimized shrink parameters seem environment depended and device depended. We did not find out an identical optimized parameter for different SLAM sensors in this research.

#### 3.2.2. Registration

The outputs of SLAMMER, NAVIS, Matterport and TLS are in their own coordinates, the registration has to be considered before the accuracy evaluation, which means the map results must be converted to identical coordinate for further comparison. Finally the map accuracy can be assessed by RMS error of the selected feature points thought Least Square Fitting (LSF) algorithm without considering the scale factor and the main equations are as below [49].

Assuming $p_{loc}=[(x_{1}^{p_{loc}},y_{1}^{p_{loc}}),\ldots ,(x_{n}^{p_{loc}},y_{n}^{p_{loc}})]$ is the feature point in SLAMMER, NAVIS, Matterport local coordinate system and $q_{\mathrm{tls}}=[(x_{1}^{q_{tls}},y_{1}^{q_{tls}}),\ldots ,(x_{n}^{q_{tls}},y_{n}^{q_{tls}})]$ is the same feature point in TLS reference coordinate system, *R* is the rotation matrix and *dθ* is the rotation angle, *t* is translation vector, the details are given in detail in following equations:

(1) $$R=⎡\begin{array}{c} \cos(d\theta )\;-\sin(d\theta )\\ \sin(d\theta )\;\;\cos(d\theta ) \end{array}⎤$$

*t* = [d*x*, *dy*] (2)

Assuming $V={\left(V_{x_{tls}},V_{y_{tls}}\right)}^{T}$ is the vector of correction in TLS’s coordinate reference system of *n* pairs of points, and the registration process is performed by minimizing the vector of correction, which can be modeled as follows:

(3) $$\min\left(V\right)=min\left(Rp_{loc}+t-q_{tls}\right)$$

Solving for rotation angle and translation vector, an error function transformed from Equation (3) is expressed in matrix format after linearization, rotation matrix and translation vector, which is shown as follows:

(4) $$\min\left(V\right)=min\left(Rp_{loc}+t-q_{tls}\right)=\min\left(B\delta X+L\right)$$

In Equation (4), *B* is the coefficient matrix, *L* is the constant vector, $\delta X={\left(dx,dy,d\theta \right)}^{T}$ is the vector of translation and rotation.

Assuming *α*_0_ is the initial rotation angle. The matrix *B* and *L* are given in detail as following equations:

(5) $$B=[\begin{array}{ccc} 1 & 0 & \left(y_{1}^{p_{loc}}\cos\alpha_{0}-x_{1}^{p_{loc}}\sin\alpha_{0}\right)\\ 0 & 1 & -(y_{1}^{p_{loc}}\sin\alpha_{0}+x_{1}^{p_{loc}}\cos\alpha_{0})\\ 1 & 0 & \left(y_{2}^{p_{loc}}\cos\alpha_{0}-x_{2}^{p_{loc}}\sin\alpha_{0}\right)\\ 0 & 1 & -(y_{2}^{p_{loc}}\sin\alpha_{0}+x_{2}^{p_{loc}}\cos\alpha_{0})\\ 1 & 0 & \left(y_{3}^{p_{loc}}\cos\alpha_{0}-x_{3}^{p_{loc}}\sin\alpha_{0}\right)\\ 0 & 1 & -(y_{3}^{p_{loc}}\sin\alpha_{0}+x_{3}^{p_{loc}}\cos\alpha_{0})\\ \vdots & \vdots & \vdots \\ 1 & 0 & \left(y_{n}^{p_{loc}}\cos\alpha_{0}-x_{n}^{p_{loc}}\sin\alpha_{0}\right)\\ 0 & 1 & -(y_{n}^{p_{loc}}\sin\alpha_{0}+x_{n}^{p_{loc}}\cos\alpha_{0}) \end{array}]$$

(6) $$L=[\begin{array}{c} x_{1}^{q_{tls}}-x_{1}^{p_{loc}}\cos\alpha_{0}-y_{1}^{p_{loc}}\sin\alpha_{0}\\ y_{1}^{q_{tls}}-y_{1}^{p_{loc}}\cos\alpha_{0}+x_{1}^{p_{loc}}\sin\alpha_{0}\\ x_{2}^{q_{tls}}-x_{2}^{p_{loc}}\cos\alpha_{0}-y_{2}^{p_{loc}}\sin\alpha_{0}\\ y_{2}^{q_{tls}}-y_{2}^{p_{loc}}\cos\alpha_{0}+x_{2}^{p_{loc}}\sin\alpha_{0}\\ x_{3}^{q_{tls}}-x_{3}^{p_{loc}}\cos\alpha_{0}-y_{3}^{p_{loc}}\sin\alpha_{0}\\ y_{3}^{q_{tls}}-y_{3}^{p_{loc}}\cos\alpha_{0}+x_{3}^{p_{loc}}\sin\alpha_{0}\\ \vdots \\ x_{n}^{q_{tls}}-x_{n}^{p_{loc}}\cos\alpha_{0}-y_{n}^{p_{loc}}\sin\alpha_{0}\\ y_{n}^{q_{tls}}-y_{n}^{p_{loc}}\cos\alpha_{0}+x_{n}^{p_{loc}}\sin\alpha_{0} \end{array}]$$

A LSF is employed to solving for rotation and translation matrix. Assuming the weight matrix in LSF is P, and a model is written as follows.

(7) $$\;\min(V^{T}PV)=min({\left(B\delta X+L\right)}^{T}P\left(B\delta X+L\right)\;$$

By finding the first derivative of δX, an equation is obtained as follows:

(8) $$\;B^{T}PB\delta X+\;B^{T}PL=0\;$$

The matrix δX can be solved through Equation (8), and δX is written as:

(9) $$\;\delta X=\;-{\left(B^{T}PB\right)}^{-1}B^{T}PL\;$$

In addition, an indicator RMS for evaluation of the accuracy of the mapping is defined as:

(10) $$\;RMS=\sqrt{\frac{\sum_{i=1}^{n}V^{2}}{n}}\;$$

## 4. Results and Discussion

### 4.1. The First Scene: The L Shape Corridor

During the field test, the Matterport sensor was installed on a tripod for easily mapping the whole corridor. In total, six survey campaigns were carried out with the Matterport. Since the distance between two neighboring scans varies, the number of scans also changes from 41 to 197 and the duration of each survey varies from one hour to more than three hours. The sensor was installed at a height of 1.8 m and leveled first before each survey. However, since the laminated floor was considered flat, the leveling of the sensor was not adjusted during the survey. The following figures illustrate the scan location for indoor mapping with its corresponding scan number of each scan.

**Figure 6:**
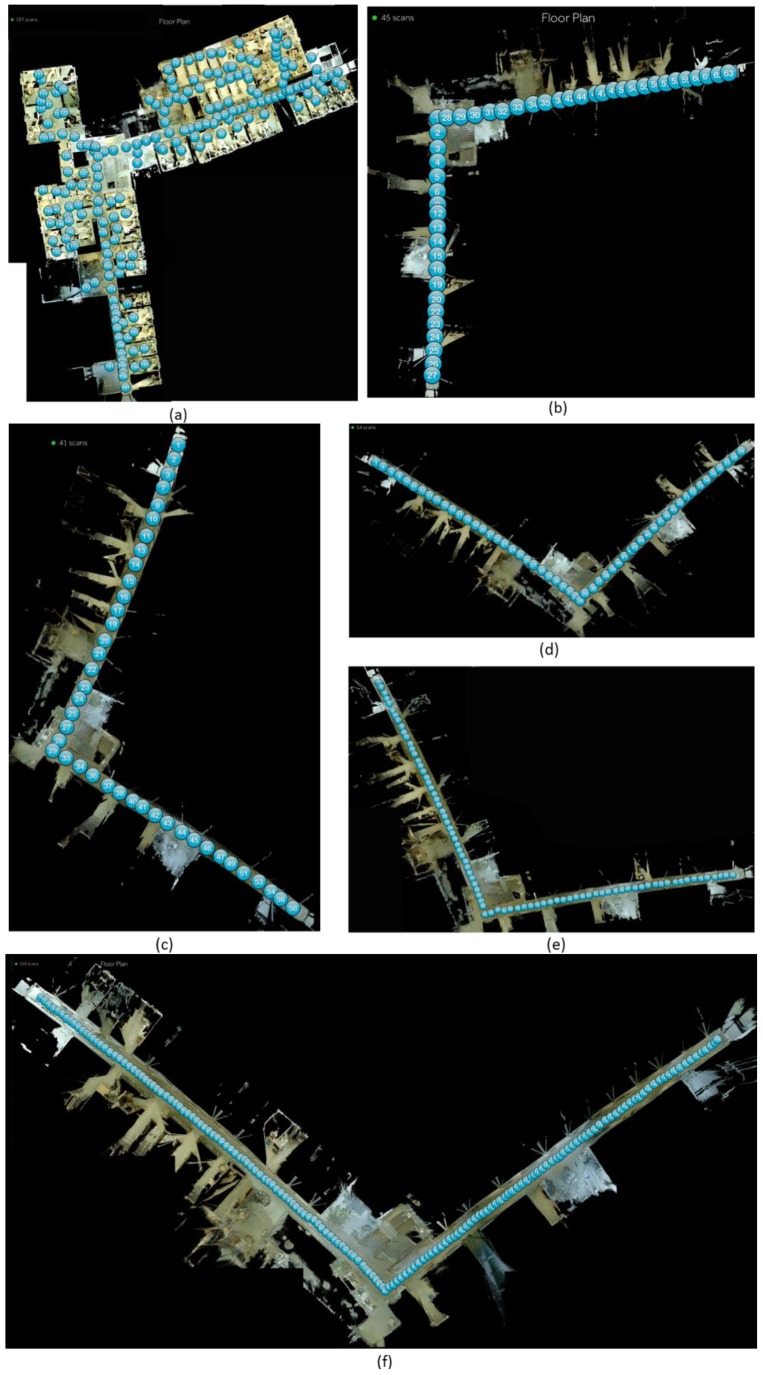
Floor map dataset generated by different setups of Matterport with positions of each scan (**a**) T1: random neighboring scan distance starting from the corner; (**b**) T2: 2 m neighboring scan distance starting from the corner; (**c**) T3: 2 m neighboring scan distance starting from the end; (**d**) T4: 1.5 m neighboring scan distance starting from the end; (**e**) T5: 1 m neighboring scan distance starting from the end; (**f**) T6: 0.5 m neighboring scan distance starting from the end.

The general description of each survey of the Matterport sensor can be found in Table 3. One thing has to be noted that, the distance between the neighboring scan is identical, due to the fail scan matching, the position of the tripod has to be slightly adjusted for a successful matching resulting in the total scan number of T2 and T3 has trivial difference. From the Table 3, following preliminary conclusions about the sensor can be drawn:

- The less the distance between neighboring scan, the more the total scan number is and the longer scan time is needed. The approximate coverage for the Matterport sensor is approximate 150 m^2^/h to 60 m^2^/h.
- The fail scan number and fail rate decrease when the distance between the neighboring scan decreases which implies that the density of scan effects the final mapping results. It has been addressed that the fail scan essentially increases the scan time.
- The manual selection of matched scans is efficient to mitigate the fail scan, however, it is only employed in a matrix measuring mode like Figure 6a presents where there is more than 1 neighboring scan to match. And it is not suitable for a linear measuring mode as T2–T6 surveys adopted where only one previous measurement can be utilized for matching.

#### 4.1.1. Matterport is a SLAM

**Figure 7:**
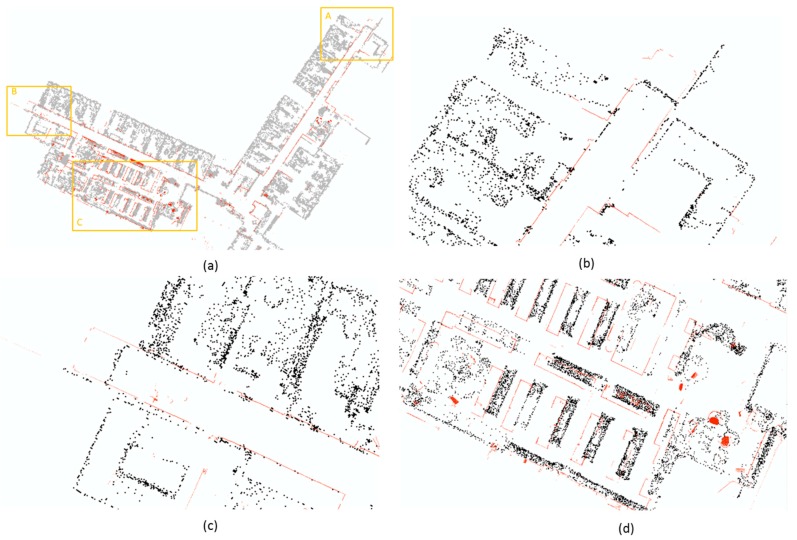
Mapping results comparison between T1 with TLS reference, where TLS reference is illustrated in red color against the black point cloud collected by Matterport (**a**) an overview comparison, (**b**) details at the end of east wing, (**c**) details at the end of south wing, (**d**) details of the open library in south wing.

To address the first aforementioned question, we compared the output of T1 with reference TLS data as Figure 7 presents. Two dataset is aligned at the corner of the two corridors where the start point of T1 is. The south corridor is aligned with the reference data. The point cloud generated by T1 is illustrated in black while the TLS reference is in dark red. Figure 7a offers an overview of the comparison. The area marked by three orange rectangles with corresponding name is enlarged in Figure 7b for area A, Figure 7c for area B, and Figure 7d for area C thus the details of the measurements can be observed. From Figure 7b,c, we can discern the difference of the end in two mapping solutions. The lengths of the mapped corridors are shorter approximate 1 m at each end in Matterport survey. While the heading estimation of the corridors is satisfied as the point cloud of the wall coincide with reference. From Figure 7d, it can be observed that map error (the difference between the TLS reference and map generated from T1) is proportional to the distance of the mapped objects to the start point. As five parallel bookshelves in the lower part of the Figure 7d presents, the further it locates from the start point, the larger mapping error is. It obviously matches the characteristics of SLAM technology. Based on the comparison carried out in this research, we can offer a positive answer to the first question.

#### 4.1.2. Mapping Trajectory Influence the Final Result

**Figure 8:**
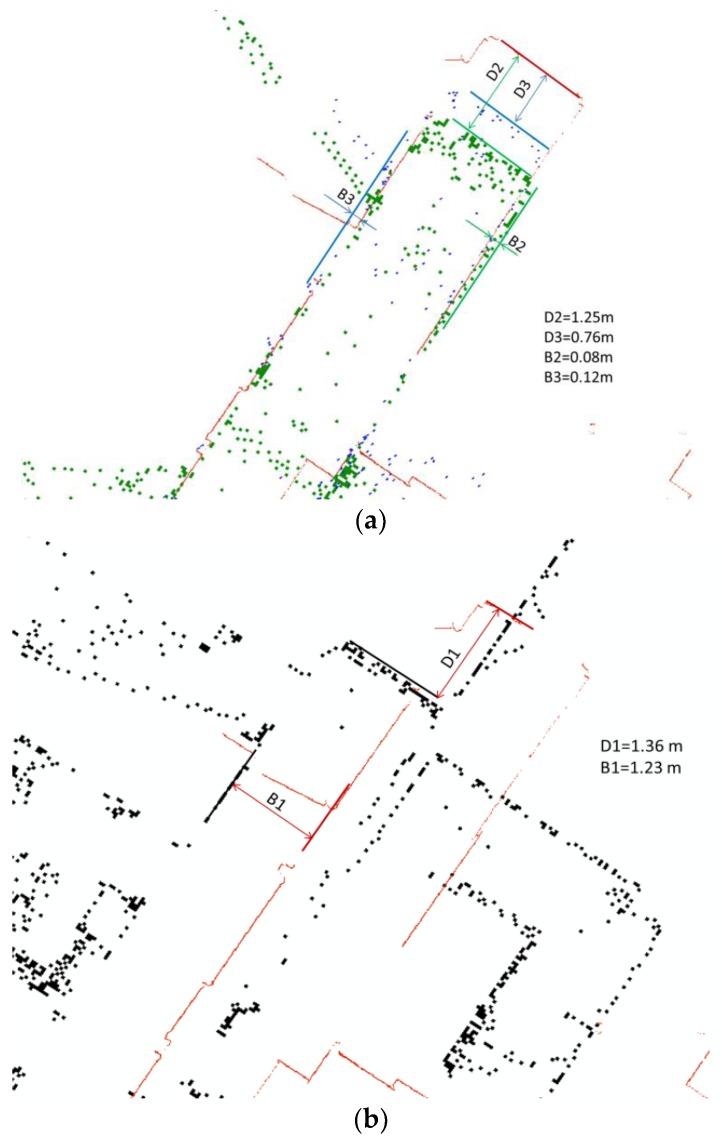
Matterport mapping results with different trajectories (**a**) 2 m mapping distance, linear trajectory and different start point; (**b**) 0.5–3 m mapping distance, matrix trajectory.

For the second question, we compared the map generated from dataset collected in T2 and T3, which has same distance between the neighboring scan with different start point: T2 started from the corner of the corridors and surveyed the east corridor first then the south corridor; T3 started from the end of south corridor, then the east corridor. T1 is also selected, because comparing with other survey, T1 is collected with “matrix” trajectory against linear trajectory. The “matrix” here implies that the data of more than two scans are utilized for matching and also the operator droved the tripod in a more detoured means. By aligning the end of south corridor, we can achieve some preliminary results such as, bias error and distance error by comparing the end of the east corridor. The bias error is the rotating error which is mainly caused by the heading estimation error of the SLAM algorithm and the distance error is travel distance error which is accumulated by the offset estimation error in continuous scan of the employed SLAM. From the bias error, the head estimation error can be estimated if the total length of the east corridor can be measured. From the reference TLS data, the length of corridor can be measured. The plus mark in bias error indicates the rotating error is clockwise and vice versa. The plus mark in distance error specifies the measured distance is longer than the reference value and vice versa. The comparisons are presented in Figure 8 and the results are summarized in Table 4. It suggests that the mapping trajectory will influence the final mapping result.

#### 4.1.3. Optimal Parameter is Feasible for Matterport Indoor Mapping

**Figure 9:**
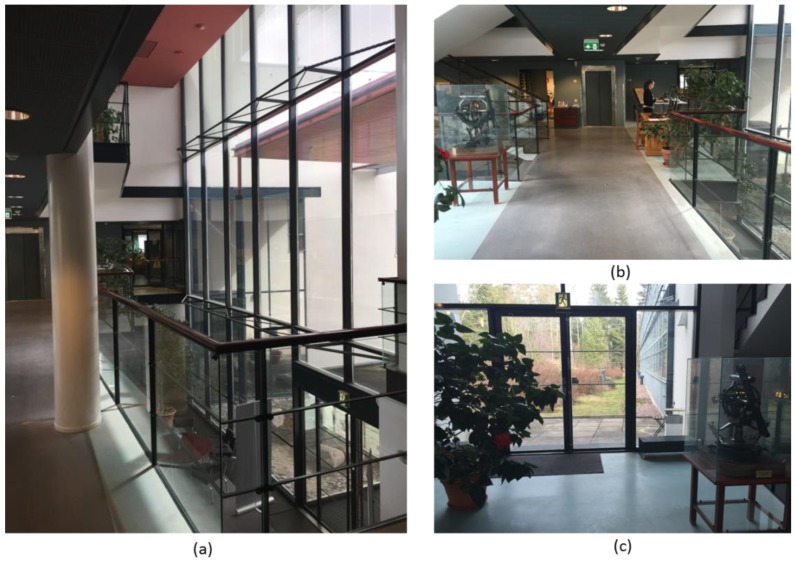
(**a**) The glass wall at the south of the corner, (**b**) the corridor of the second floor with various glass made infrastructure (**c**) the entrance at the north of the corner.

For the third question, we compared the mapping results of T2–T6 which mapped the corridor at linear mode with different distance between neighboring scan. The results are summarized in Table 5. It can be observed from the Table 5 the distance error decreases when the distance between neighboring scan decrease. On another words, the dense the measurement is, the better length accuracy can be anticipated for Matterport measurement. However, the head estimation accuracy doesn’t follow the same trend. There are several reasons we suspect might cause the unsatisfied head estimation for Matterport surveys:

- The corner area of the corridor which contains a lot of contracture made of glass as Figure 9 presents. The reflection characteristics of the surface of glass made objects might bias the head estimation of the matching processing.
- No texture is attached with glass, with less feature, the accuracy of the matching processing might degrade.
- The 3-floor height glass wall in south front of the corner and the glass entrance of north front of the corner (Figure 9a) will introduce direct sunlight during the survey campaigns which might mislead the heading estimation error, and this should be avoided based on user manual recommendation. It is impractical to use large shades or drapes to block all nature light source during the survey.

However, there is still an optimal setup configuration could be recommended for indoor corridor mapping: 1 m between neighboring scans.

### 4.2. The Second Scene: An Open Style Library in FGI

Since the nature and specification of the ranging sensors are diverse, in Table 6, we list the number of points utilized in the accuracy evaluation of the second scene in each case. It can be observed that the point cloud density achieved using Matterport is approximate two order of magnitude lower compared with the laser scanning technology.

#### 4.2.1. Accuracy Evaluation with Interactively Selected Feature Points

**Figure 10:**
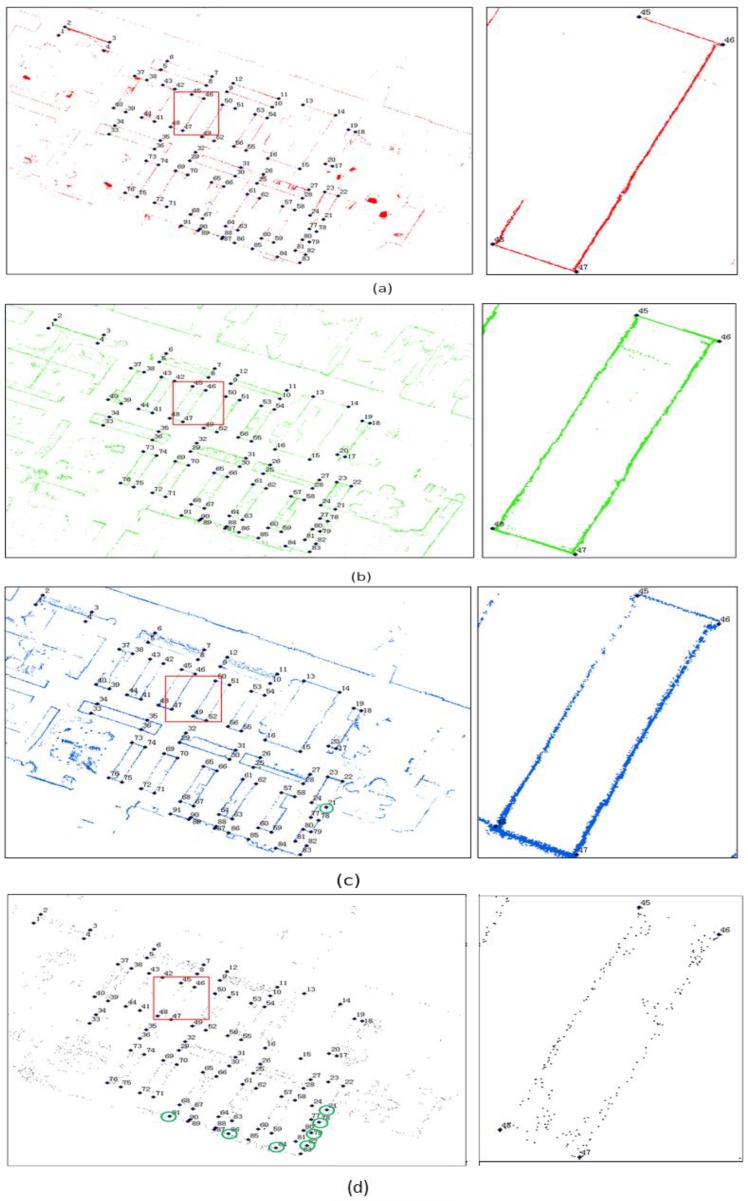
(**a**) TLS reference map and the interactively selected key points; (**b**) transformed SLAMMER map result with interactively selected key points by LSF algorithm (**c**) transformed NAVIS map result with interactively selected key points with LSF algorithm (**d**) transformed Matterport map result with interactively selected key points with LSF algorithm.

Figure 10 shows the indoor map collected by TLS, and the transformed SLAMMER, NAVIS and Matterport. By comparing the zoom-in-images of each mapping system, it is obvious that line features in NAVIS and Matterport map results are remarkably noisier than those in SLAMMER and TLS. The profile of a bookshelf is clear in TLS and SLAMMER and they represent almost identical rendition; NAVIS represents moderate rendition capability with considerable noise: the profile is wider than its counterparts generated by the high accurate laser scanner, and the point cloud of the corners of bookshelf are scattered because the applied Hokuyo laser scanner is a big footprint scanner; The point cloud generated by Matterport was considerable dispersed comparing with other three solutions and the linearity of the profile is worse than that of the SLAMMER and the NAVIS, and the corner is also too ambiguous to be detected. The explanations of the different profile quality are: (1) both TLS and SLAMMER adopt small footprint millimeter accuracy laser, the smaller the footprint is, the less measurement noise introduced by the corner; (2) as SLAMMER maps the indoor in a dynamic mode, the profile contains more noise, introduced by vibration of the system during scanning, and fine scale scan match errors; (3) the NAVIS use large-footprint laser scanner with centimeter ranging accuracy, it will introduce more noise especially in long range measurement; (4) the Matterport generated considerable sparser point cloud in comparison to the three other methods.

Residuals and RMS errors of the selected feature points of the three investigated indoor mapping technologies are listed in Table 7. There are in total 91 feature points of corners picked out from the TLS reference map, but not all of them were used in the evaluation. This was, because some of the corners were missing or not clear enough in the generated SLAM maps for reliable feature detection (being in part an indicator of the performance of a particular system). Therefore, 91, 90 and 84 feature points were found from SLAMMER, NAVIS and Matterport mapping systems for evaluation, respectively. The only undetected feature point of NAVIS output is point No. 21 (circulated in Figure 10c) which locates on the corner of a narrow corridor, the chairs on the narrow corridor blocked the large footprint laser pulse during the mapping. However, for SLAMMER, the small footprint laser can overcome it. All undetected feature points of Matterport (circulated in Figure 10d) output locate at the bottom corner due to the low illumination. The RMS errors were 20, 37 and 42 mm correspondingly. It means that SLAMMER with FARO LiDAR yields to the best mapping accuracy of the three, NAVIS gives reasonable accuracy and Matterport shows similar, but slightly less accurate performance.

#### 4.2.2. Accuracy Evaluation with Feature Points Selected by MBRs

**Figure 11:**
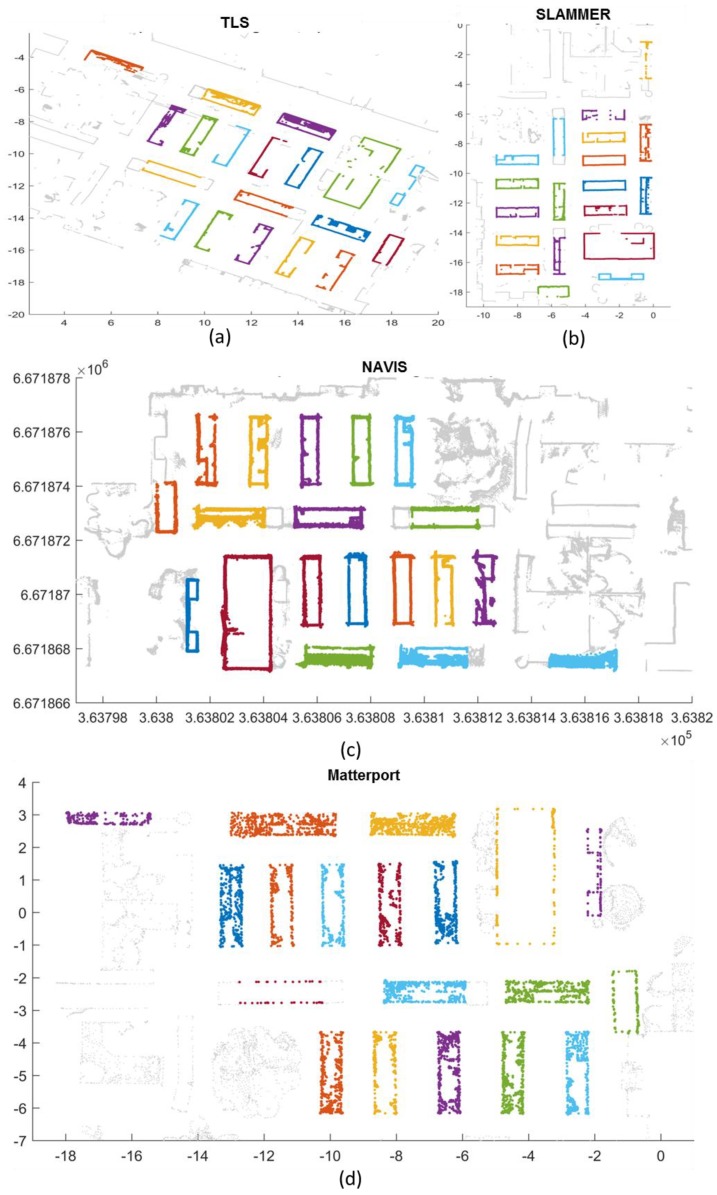
Segmentation results of mapping data from (**a**) TLS reference, (**b**) SLAMMER (**c**) NAVIS and (**d**) Matterport in local coordinate.

The segmenting results of the data from the three different mapping technologies and the TLS data are presented in Figure 11. Each “areas of interest” with interactive segmentation are illustrated in different colors against the gray background point cloud. It can be observed that except for one glass book cabinet in Matterport point cloud; most of the “area of interest” can be detected.

**Figure 12:**
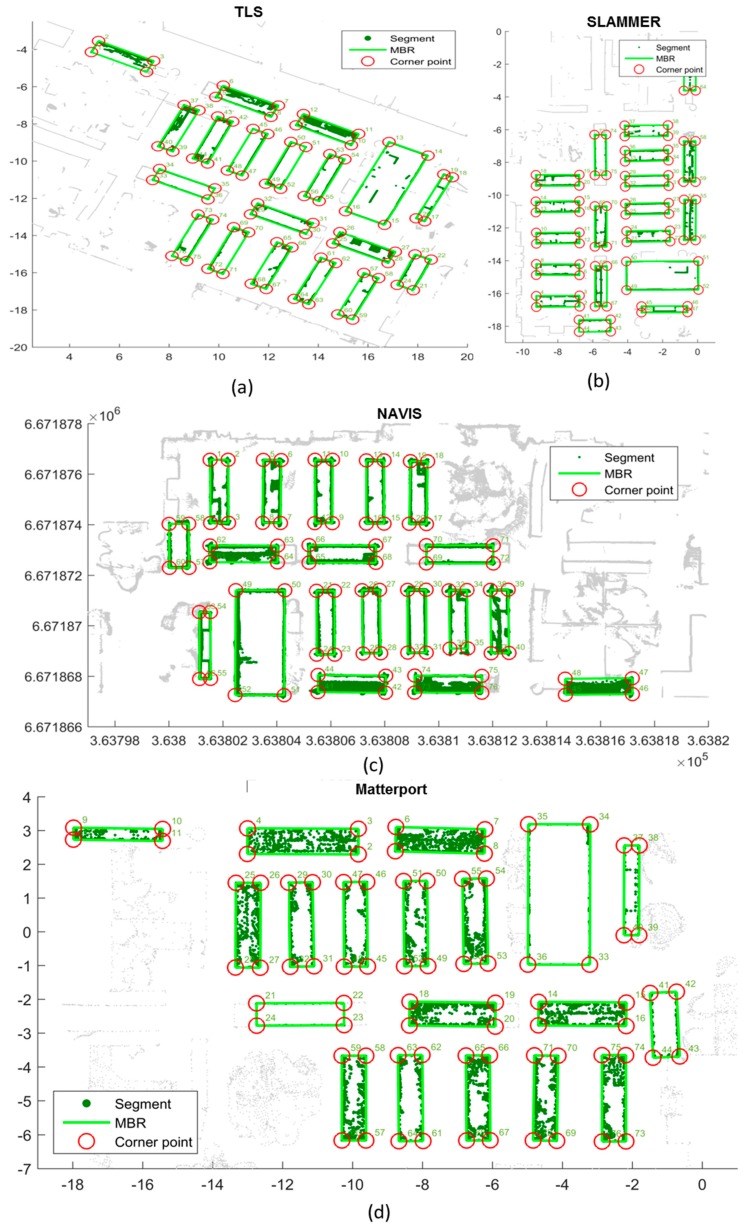
Feature points extracted from MBR of (**a**) TLS, (**b**) SLAMMER, (**c**) NAVIS, and (**d**) Matterport.

**Figure 13:**
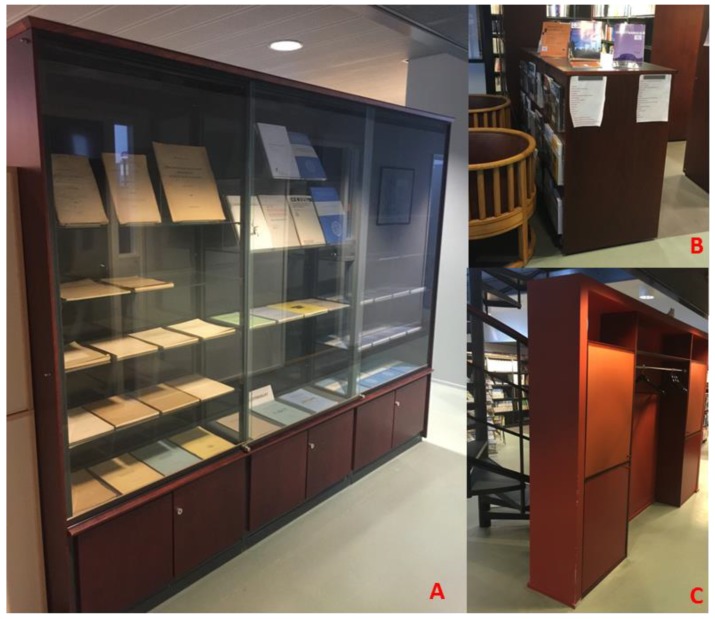
(**A**) Glass book cabinet misdetected by the Matterport sensor, (**B**) a bookshelf behind chairs; (**C**) a wardrobe behind stairs.

The feature point extraction results of the three different systems and TLS data are also presented in Figure 12, and the TLS data is shown in the background for illustration purposes. In Figure 12, the extracted rectangles are illustrated in light green color. The corners of each extracted rectangle are marked with a red circle with a unique number. From the TLS reference data, in total 76 reference points are extracted with MBRs, which means 19 rectangular objects were successfully extracted, among them, there are three book cabinets with glass front door, 14 book shelfs, a toilet room and a wardrobe. The detection rate of feature points are 100%, 97.3% and 94.7% respectively shown in Table 8. For SLAMMER point cloud, all feature points were correctly extracted; while for NAVIS, two feature points (marked as 35 and 36 in Figure 13d) were undetected by the proposed MBRs. However in Matterport case, one glass book cabinet cannot be detected; it is clear that in Matterport point cloud segmentation Figure 12d, one glass book cabinet (A in Figure 13) cannot be correctly detected. Even though total 76 feature points are extracted, and among them, at least four feature points are mismatched (marked as 9, 10, 12, 13 in Figure 11d) due to its positioning error larger than a preset error threshold (0.1 m). During the Matterport measurement campaign, it was also observed that the consistence of lighting/illumination situation, the appearance of the dynamic objects as well as the glass might extensively interfere the modeling result. Another restriction for Matterport might be the terrestrial scan mechanism: in order to achieve a detailed 3D model of the indoor complex structure; extra scans might be needed during the measurement to model the wardrobe (B in Figure 13) behind the stair and the bookshelf (C in Figure 13) behind the chairs. The operation time might also be a restriction for large area indoor mapping application because the maximum distance between two neighboring scans is 3.7 m (12 feet). It spent 2 h to scan FGI library with 65 scans against several minutes for NAVIS and SLAMMER (SLAMMER additionally requires few minutes for initializing IMU).

Table 9 summarizes the advantages and disadvantages of three indoor mapping technologies investigated in the research based on the accuracy evaluation results. It can be preliminary concluded that

(1) three SLAM-based indoor mapping technologies can all generate centimeter accuracy mapping result within a medium size managed indoor environment;
(2) indoor mapping accuracy can be promoted by increasing the range accuracy of the adopt LiDAR or decreasing the footprint size of laser beam. by comparing the results of the SLAMMER and the NAVIS; the explanation is with more accurate measurement, the matching accuracy between continuous scan can be improved, and with smaller footprint laser beam, the measurement noise will also decrease, which results in the better mapping result;
(3) Matterport is a reliable tool to generate 3D indoor model. However, extra labor is needed to filter out the mismatched feature points to produce a credible 2D map. Also the mapping accuracy is slightly worse than other two LiDAR based solutions.

## 5. Conclusions

The three studied SLAM-based indoor mapping technologies can offer centimeter mapping accuracy in complex indoor environments. The results indicate that by utilizing a more accurate LiDAR sensor, SLAM-based indoor mapping accuracy can be improved compared to the generally produced by industrial grade low cost sensors. There is a broad selection of fresh sensors on the market falling between the two grades investigated in this study featuring moderate prices.

However, restricted by its terrestrial scan mechanism, range limit and sensor resolution, the density of point cloud produces by the Matterport is considerably lower than those of the two other technologies. Some feature points could not be extracted either by the interactive or MBR selection. Thus extra interactive examining might be necessary to rectify the undetected results to generate the 2D indoor map. The mapping accuracy of Matterport is slightly lower than other two LiDAR-based methods.

## Figures and Tables

**Table 1:** Technical specification of SLAMMER.

**Laser Scanner (120S/X330)**	
Range	150 m/300 m
Wavelength	905 nm/1550 nm
Field of View	305 degrees
Max. measurement rate	976k point/s
Profile rate	30–97 Hz
Angular resolution	0.157 mrad
Range error	±2 mm
Ranging noise	0.95–2.2 mm
**IMU**	
Gyro Technology	Fiber Optics Gyros
Data Rate	200 Hz
Gyro Bias	<1.0 deg/h
Angular random walk	0.05 degree/
Attitude Accuracy	Roll:0.005 degree
	Pitch: 0.005 degree
	Heading 0.008 degree
Time accuracy	20 ns
Weight	4.25 kg + 5.2 kg + 5.2 kg + tablet and frame

**Table 2:** Technical Specification of NAVIS.

**Laser Scanner**	
Range	0.1–60 m
Wavelength	905 nm
Field of View	270°
Measurement rate	43,200 points/s
Profile rate	40 Hz
Angular resolution	0.25°
Range error	±30 mm
Ranging noise	10 mm
**IMU**	
Gyro Technology	MEMS
Data Rate	100 Hz
Gyro Bias Stability	10 degree/h
Angular Random Walk	3
Velocity Random Walk (VRW)	0.12
Attitude Accuracy	Roll: 0.3 degree
	Pitch: 0.3 degree
	Heading 1 degree
Total Weight	270 g + 58 g + laptop and frame

**Table 3:** General description of dataset collected for the comparison.

Surveying Campaign	Distance between Neighboring Scan	Start Point	Total Scan	Fail Scan	Fail Rate	Scan Time
T1	0.5–3 m	Corner	197	0 *	0%	195 min
T2	2 m	Corner	41	17	29.3%	95 min
T3	2 m	End	45	18	28.6%	102 min
T4	0.5 m	End	159	0	0%	148 min
T5	1 m	End	81	2	2.4%	87 min
T6	1.5 m	End	54	5	8.5%	60 min

* Manual selection of matching scans is needed.

**Table 4:** Comparison of Matterport output with different mapping trajectory.

Surveying Campaign	Distance between Neighboring Scan	Start Point	Bias Error	Heading Estimation Error	Distance Error
T2	2 m	Corner	0.08 m	0.11°	−1.25 m
T3	2 m	End	−0.12 m	−0.17°	−0.76 m
T1	0.5–3 m	Corner	−1.23 m	−1.76°	−1.36 m

**Table 5:** Matterport mapping results with different setting.

Surveying Campaign	Distance between Neighboring Scans	Start Point	Bias Error	Heading Estimation Error	Distance Error
T2	2 m	Corner	0.08 m	0.11°	−1.25 m
T3	2 m	End	−0.12 m	−0.17°	−0.76 m
T4	0.5m	End	1 m	1.43°	−0.15 m
T5	1 m	End	0.32 m	0.46°	0.15 m
T6	1.5 m	End	0.80 m	1.15°	−0.70 m

**Table 6:** The number of point for the three different indoor mapping solutions utilized for the accuracy evaluation.

	SLAMMER	NAVIS	Matterport	TLS
Point	4.29 M	1.01 M	12,098	3.7 M

**Table 7:** Accuracy assessment results for the interactive selected feature points of the library.

	TLS	SLAMMER	NAVIS	MATTERPORT
RMSE (m)	-	0.020	0.037	0.042
Feature points	91	91	90	84
Detection rates	-	100%	98.9%	92.3%

**Table 8:** Accuracy assessment results for feature points selected by MBR.

	TLS	SLAMMER	NAVIS	MATTERPORT
Extracted feature points	76	76	76	76
Effective feature point	76	76	74	72
Detection rate	-	100%	97.3%	94.7%
RMSE (m)	-	0.017	0.032	0.047

**Table 9:** Comparison of SLAMMER, NAVIS and Matterport.

	SLAMMER	NAVIS	Matterport
Effective Range	150 m	0.1–60 m	12 feet between scans
IMU	Tactical-grade IMU	Consumer-grade IMU	None
Mobility	Yes	Yes	No
Texture	No	No	Yes
Measure with disturbance	Yes	Yes	No
Cost	High	Low	Low
Real Time Performance	Yes (real time mode with lower mapping accuracy)	Yes	No
Scanning time	Short	Short	Long
Range Sensor	FARO Focus3D 120S	Hokuyo UTM-30LX-EW	Kinect type depth camera
Footprint size (at 10 m)	4.2 mm	>130 mm	-
Range Accuracy	2 mm	3 cm	-
Initialization outdoor	Yes	No	No
Synchronization	Hardware	Software	-
Dynamic Object Filter	No	Yes	No
Point Cloud Density	High	Medium	Low
Data Collecting Efficiency	High (several minutes for FGI library)	High (several minutes for FGI library)	Low (2 h for FGI library)
Weight	High	Low	Low
Power Consumption	High	Low	Low
Operator	trained operator	trained operator	untrained operator
